# Annotations

**Published:** 1903-03-21

**Authors:** 


### ANNOTATIONS.

### The Practitioner and the Public.

AMONG the many unpaid responsibilities of the general practitioner is that of recommending patients for admission to hospitals and convalescent homes. Some doctors have a real comprehension of what this duty means—that it is a question not only of the disease but of all the circumstances of the patients. It is not a question of social position only, for occasionally one finds a poor professional man who is less able than many an artisan to pay for special treatment, nor indeed can the question of fitness for receiving a medical charity be decided by any one feature of the case. Some doctors feel to the full their responsibility in this matter; some do not. For example, an appeal came lately to a gentleman who was known to have some influence in hospital matters from a sufferer who was indeed crippled with rheumatism and might have been much benefited by a visit to some place where suitable baths and other treatment could be obtained, but who was the owner of a flourishing boarding-house, and had besides a husband and son who were earning a decent income. Her doctor had given his opinion that the case would be benefited by the change of air and special treatment, and took into account no other point in the person's condition. Such incidents make it an imperative duty for those who have hospital tickets to give, or can in any way obtain admission to medical institutions for those they recommend, to investigate every appeal which is brought before them, and thus to give their charity wisely. It might be supposed that this being obvious, medical men would welcome such inquiries into the cases which they recommend. Unfortunately they occasionally do not. "Do you think I would recommend a case that did not require hospital treatment?" is the indignant query that often meets the charitable person or his representative who makes a few inquiries into the general circumstances of the case for which help is asked. But the inquiry implies no doubt of the *bonâ fides* of the doctor. One would be quite willing to take his opinion on the pathological aspects of the case. But the amount of accommodation available in our hospitals and convalescent homes is limited, and — in theory, at least—we all wish that those who need it most should benefit by our medical charities. One wants to know also, before giving charity, if the patient or his friends cannot pay for the relief needed. If so, they ought to do it. An appeal for help for our hospitals is often met by a protest that our medical charities are abused, and that gratuitous treatment is given to those who could well afford to pay for it. Those who have to do with hospitals know that this is too often true, and are as anxious as any that the reproach should be removed. It is certainly in the interests of the general practitioner that no one should get free treatment who can afford to pay for it, and it is strange that the *esprit de corps* of the profession is not sufficiently strong to do more towards securing this. An inquiry made into the circumstances of a case is in the interest of the doctor who has attended it, or at least in the interests of the struggling practitioner who works hard for a very modest living in our poorer districts, and should not be injured by the existence of medical charities, and it should be welcomed and aided by every medical man. At present such inquiries are made only privately and unofficially. Whether, as our hospitals become more organised into one large network of charity, they should not be made officially, and as a matter of course, is a question for the future to decide. Official inquiries, because they are official, and therefore a matter of course, at least give no offence to anyone. And assuredly inquiry is necessary. We do not want to bind charity with fetters of red tape; but on the other hand it should not be captured by meanness and greed, as it sometimes is at present. By receiving investigations, made in a kindly spirit, in a spirit as kind, medical men would help both the hospitals and themselves.

### "To Stay at Home is Best."

As the early travellers return from the Riviera and tell their tales of bitter winds and insufficient means of heating their rooms, one feels inclined to ask them why they so determinedly go abroad year after year and never try some of the warmer spots in England. This year, at least, it has been admittedly pleasanter at home than in the South of France, and for many people it is more convenient, even when on holiday, to be within easy reach of their permanent home. Good hotels and boarding-houses are to be found in almost every English watering place, the prices are less exorbitant than those which the foreigners charge, and the little comforts which English folk desire are more easily attainable. And it is probable that the sanitary record of the place will be more satisfactory than that of many foreign towns. We have before us, for example, the health record of Hastings for the last six months. As Hastings is a place where consumptives go, it is to be expected that both in public institutions and in private houses and hotels there will be the deaths of invalids to swell the mortality lists, and these deaths should not be debited to the borough itself. Yet, even including these, the report shows a comparatively small record of deaths of young persons. A very large proportion are deaths of persons over 60 years of age—deaths which may fairly be regarded as natural. The death-rate among young children—the most delicate class in every community—is small and is steadily decreasing. The death-rate for the last quarter of last year was 14·08 per thousand, and that for the quarter which ended September 30th, only 11·43 per thousand. As the average death-rate for England and Wales is 16·3 per thousand, it is clear that the health standard of Hastings, even during the worst quarter of the year, is above the general average, and stands thus high even though we include the deaths of those who went there in an almost moribund condition. That it is a pleasant place all who have been there know—sheltered from bleak winds and catching more than its share of sunshine. Moreover, it is within easy reach of London, which is surely an advantage. The long journey to and from the continental health-resort often undoes all the good that the place itself may do. And after all, it is pleasanter, if one is something of an invalid and is alone, as some invalids must be, to hear one's native tongue spoken all round, and to be surrounded by frank English faces. When one's health is feeble "to stay at home is best"—at any rate as near home as one can find a sufficiently genial climate.

